# Gharial (*Gavialis gangeticus*) conservation in Bardia National Park, Nepal: Assessing population structure and habitat characteristics along the river channel amidst infrastructure development

**DOI:** 10.1002/ece3.10661

**Published:** 2023-11-07

**Authors:** Bijaya Dhami, Tek Maraseni, Kanchan Thapa, Nishan K. C., Sanskar Subedi, Shreejan Gautam, Santosh Ayer, Erin Bayne

**Affiliations:** ^1^ Department of Biological Sciences University of Alberta Edmonton Alberta Canada; ^2^ University of Southern Queensland Toowoomba Queensland Australia; ^3^ WWF Nepal Kathmandu Nepal; ^4^ Institute of Forestry Pokhara Campus Tribhuvan University Pokhara Nepal; ^5^ College of Natural Resource Management (CNRM) Agriculture and Forestry University Katari Nepal

**Keywords:** Gharials, logistic regression, mid‐river depth, occupancy, river width, sex biased, water temperature

## Abstract

Nepal initiated numerous hydropower and irrigation‐related infrastructure projects to enhance and promote green energy, water security, and agricultural productivity. However, these projects may pose risks to natural habitats and the well‐being of aquatic fauna, leading to significant effects on delicate ecosystems. To understand these potential impacts, it is crucial to gather reliable baseline data on the population status and habitat characteristics of species. This study specifically focuses on Gharials (*Gavialis gangeticus*), a critically endangered species. We recorded data on pre‐determined habitat variables at stations spaced 500 m apart along the two major river streams of Bardia National Park, as well as at locations where Gharials were sighted between February and March 2023. We used binary logistic regression with a logit link function to investigate the habitat characteristics related to the occurrence of Gharials. The presence/absence of Gharials at sampling points served as the dependent variable, while 10 other predetermined variables (ecological variables and disturbance variables) served as independent variables. Our study recorded 23 Gharials, comprising 14 adults, six sub‐adults, and three juveniles, with a sex ratio of 55.56 males per 100 females. Most individuals (83%) were found basking. Among the 10 habitat predictors, three variables (mid‐river depth, river width, and water temperature) were significantly correlated (*p* < .05) with the probability of Gharial occurrence. The model shows that Gharial detection probability increases with greater mid‐river depth and width and lower water temperature. This study establishes a population baseline for Gharials within the river system before the construction of large infrastructure projects, such as dams and irrigation canals. It also recommends continuous monitoring of Gharial populations after water release and/or diversion to evaluate the impact of large infrastructure projects on the population and their associated habitat characteristics. This will help enable more informed and targeted conservation efforts.

## Graphical abstract

Critically Endangered Gharial basking along the banks of Babai River in Bardia National Park. Increase in the river disturbance is critical for survival of Gharial in the river system of Nepal.

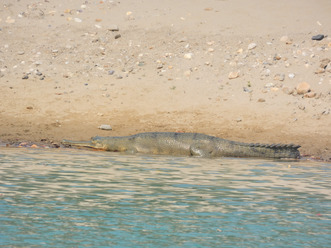

## 1 INTRODUCTION

Freshwater ecosystems, covering only 1% of the Earth's surface, host a third of all vertebrate species worldwide (Balian et al., 2008). However, the persistent of intense anthropogenic pressures on freshwater ecosystems worldwide have placed the rich biodiversity within them in jeopardy (Dudgeon et al., 2006; Reid et al., 2019), with a heightened focus on megafauna species. These larger members of their taxonomic groups face a significantly greater risk of population declines and potential extinctions compared to their smaller counterparts (He et al., 2018). This situation is expected to persist or worsen in the coming years (He et al., 2019). Main threats encompass excessive exploitation, the erection of dams, habitat deterioration, pollution, and the incursion of invasive species (He et al., 2017). For instance, dams not only disrupt the connectivity of river channels but also exert significant impacts on the overall composition of riverine landscapes (Devkota et al., 2020; Nilsson et al., 2005; Nilsson & Berggren, 2000). River‐dwelling habitat specialists encounter imminent and direct threats, including elevated harvesting pressures, constrained foraging opportunities, and the depletion of aquatic environments due to factors such as disrupted channel connectivity, altered flow regimes, and water extraction projects (Vashistha et al., 2021). If the impacts stemming from freshwater infrastructure developments, such as the construction of dams and irrigation canals, are left unattended, they have the potential to trigger declines in species populations, habitat fragmentation, and localized extinctions [e.g., Indus River dolphins (Braulik et al., 2014)] and regional extinction [e.g., Gharial in the Indus (Lang et al., 2019)]. Thus, establishing baseline estimates for key biological parameters such as population size, distribution range, and habitat characteristics is however critical for directing conservation planning efforts towards threatened and rare taxa (Jeliazkov et al., 2022; Sutton et al., 2023), particularly those that are critically endangered (Dhami et al., 2023). Current conservation measures are inadequate in protecting freshwater habitats and biodiversity as these ecosystems are typically not a priority in conservation management plans and actions (Abell et al., 2017; Collen et al., 2014; Darwall et al., 2018; Harrison et al., 2018; Pant et al., 2021).

**FIGURE 1:**
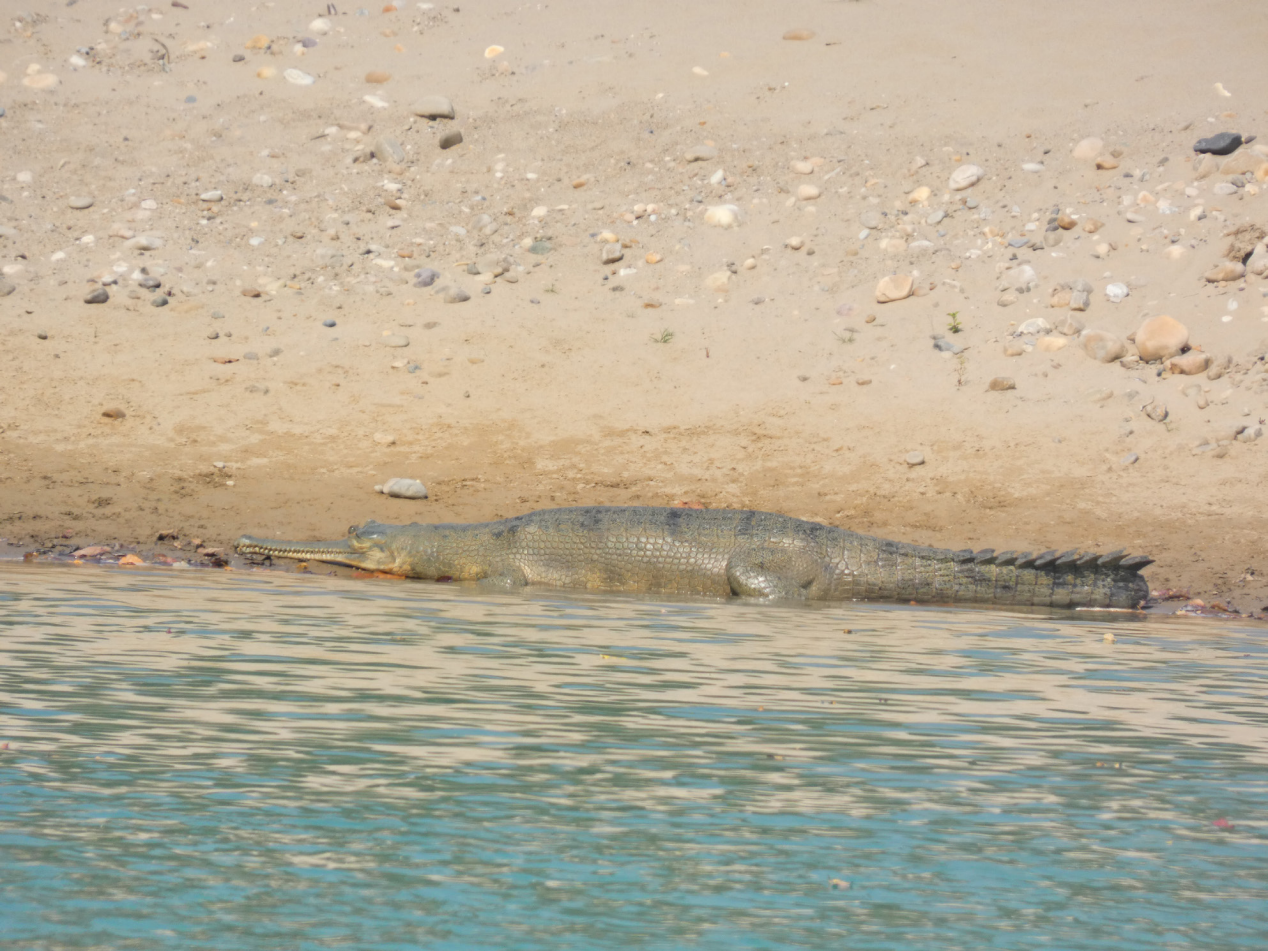
Gharial observed basking in the riverbank of Babai River stream.

Nepal has experienced a greater loss of freshwater species compared to terrestrial species, with a nearly two‐fold difference over the past five decades (WWF, 2016). Gharial (*Gavialis gangeticus*; see Figure 1) is a notable example among the freshwater reptiles at high risk of facing a rapid decline in Nepal's freshwater ecosystems. It is the only extant species of the family Gavialidae (Maskey, 1989) and is listed as critically endangered on the Red List of the International Union for Conservation of Nature (Lang et al., 2019) and appended in Appendix I of the Convention on International Trade in Endangered Species of Wild Fauna and Flora (CITES, 2021). Similarly, the Government of Nepal has listed in Schedule‐I of the protected species under the National Parks and Wildlife Conservation Act 1973 (GoN, 1973) given its conservation status in the country.

Prior to the 1940s, Gharials thrived in significant numbers, estimated between 5000 and 10,000 individuals, and inhabited major river systems spanning from the Indus in Pakistan to the Gangetic floodplains of India, Nepal, Bangladesh, Bhutan, and Myanmar's Irrawaddy (Lang et al., 2019; Whitaker et al., 1974). By the early 1970s, they had vanished from about 80% of their historic range and experienced a staggering 95% decline in population size (Lang et al., 2019), leading to their recognition as a top priority EDGE (Evolutionary Distinct and Globally Endangered) reptile species (Gumbs et al., 2018). Presently, Gharials are limited to isolated areas in Nepal and India, with an estimated population of 300–900 breeding adults in the wild (Lang et al., 2019). In Nepal, the once‐thriving Gharial populations in the Koshi and Mahakali rivers became extinct, leaving only remnant populations in the Narayani‐Rapti River system of Chitwan National Park (CNP) and the Karnali and Babai rivers within Bardia National Park (BNP) (Lang et al., 2019). The current population of the Rapti and Babai Rivers are comprised of species that were introduced from the Narayani River in 1985 and 1990, respectively. Recognizing the critical need for conservation, the government of Nepal, in collaboration with the Smithsonian Institute and the Frankfurt Zoological Society, established the Gharial Conservation and Breeding Center (GCBC) in Kasara, CNP in 1978 (Ballouard et al., 2010). This effort was initiated during a period of rapid Gharial population decline when there were less than 81 individuals recorded, and hatchling survival rates in their natural habitat were less than 1% (DNPWC, 2018).

As per the latest census of 2016, the total population of Gharials in Nepal is estimated to be 198 individuals (Acharya et al., 2017). This marks a gradual increase in the population of Gharials since the previous surveys conducted in 2011 and 2013, which recorded 102 and 124 Gharials, respectively (Acharya et al., 2017). The increase in the Gharial population has been credited to the implementation of ex‐situ conservation measures and in‐situ nesting success (Acharya et al., 2017; Bashyal et al., 2021). During an opportunistic visit in mid‐June 2019, Bashyal et al. (2021) made a notable discovery at Dhanuse: three hatched nests with an estimated 100 hatchlings nearby. According to the most recent national survey, the Narayani River hosts a population of 84 Gharials, while the Rapti River is home to 82 individuals. In addition, the Babai River supports a population of 31 Gharials, while the Karnali River currently has only one individual remaining (DNPWC, 2018). Despite the increase in the overall population, the number of adult Gharials has remained low and skewed towards females (Acharya et al., 2017). Due to their low population and the existing threats, it has become essential to conduct regular periodic monitoring of Gharials. The Government of Nepal has been systematically monitoring Gharials every 2 years since the initiation of the Gharial Conservation Action Plan in 2018, employing the direct count method (DNPWC, 2018). This enables conservation efforts to be implemented promptly to prevent any further decline in their population.

Further, it is important to recognize that infrastructure development is a significant cause of habitat disturbance and loss worldwide (Laurance et al., 2015); however, despite its far‐reaching consequences, infrastructure development has not received as much attention as other drivers of biodiversity decline, such as agriculture (Pant et al., 2021; Simkin et al., 2022). The Government of Nepal (GoN) has initiated two large‐scale projects in western Nepal. The Bheri Babai Diversion Multipurpose Project (BBDMP), which involves constructing an inter‐basin water transfer system (Bheri River to the Babai River). This transfer of water, which amounts to 40 cubic meters per second, will enable irrigation of over 51,000 hectares of agricultural land in the Bardia and Banke districts. The project will also generate 46 MW of hydropower (GoN, 2023). Rani Jamara Kulariya Irrigation Scheme (RJKIS), which aims to develop canal system to improve irrigation water delivery to, and management, in the command area of 38,300 ha of Sudurpaschim Province, Nepal (GoN/DWRI, 2023). Both BBDMP and RJKIS have been labeled as “Projects of National Pride” by the GoN despite their adverse effects on aquatic ecosystems and their functions (Das, 2006; Lakra et al., 2011; Renöfält et al., 2010). These also include both upstream and downstream impacts on habitat for aquatic communities such as fish and reptiles, and water quantity and quality (Dudgeon, 2000; Pelicice et al., 2015).

The primary objective of this study is to meticulously establish a comprehensive baseline dataset encompassing critical information pertaining to the Gharial population status and the ecological characteristics of their habitats within the Karnali and Babai Rivers. This endeavor not only addresses the current knowledge gap but also lays a solid foundation for future research endeavors in the field of Gharial conservation and riverine ecosystem management, especially addressing potential impacts of large infrastructure development, such as irrigation projects. The insights from this study can aid in identifying areas vulnerable to infrastructure development and designing mitigation measures to minimize negative impacts on Gharials and other threatened species, such as freshwater dolphins, as well as their habitat. Ultimately, this study can contribute to shaping environmentally sustainable infrastructure plans that prioritize conservation objectives, facilitating more informed and responsible development decisions.

## 2 MATERIALS AND METHODS

### 2.1 Study area

**FIGURE 2:**
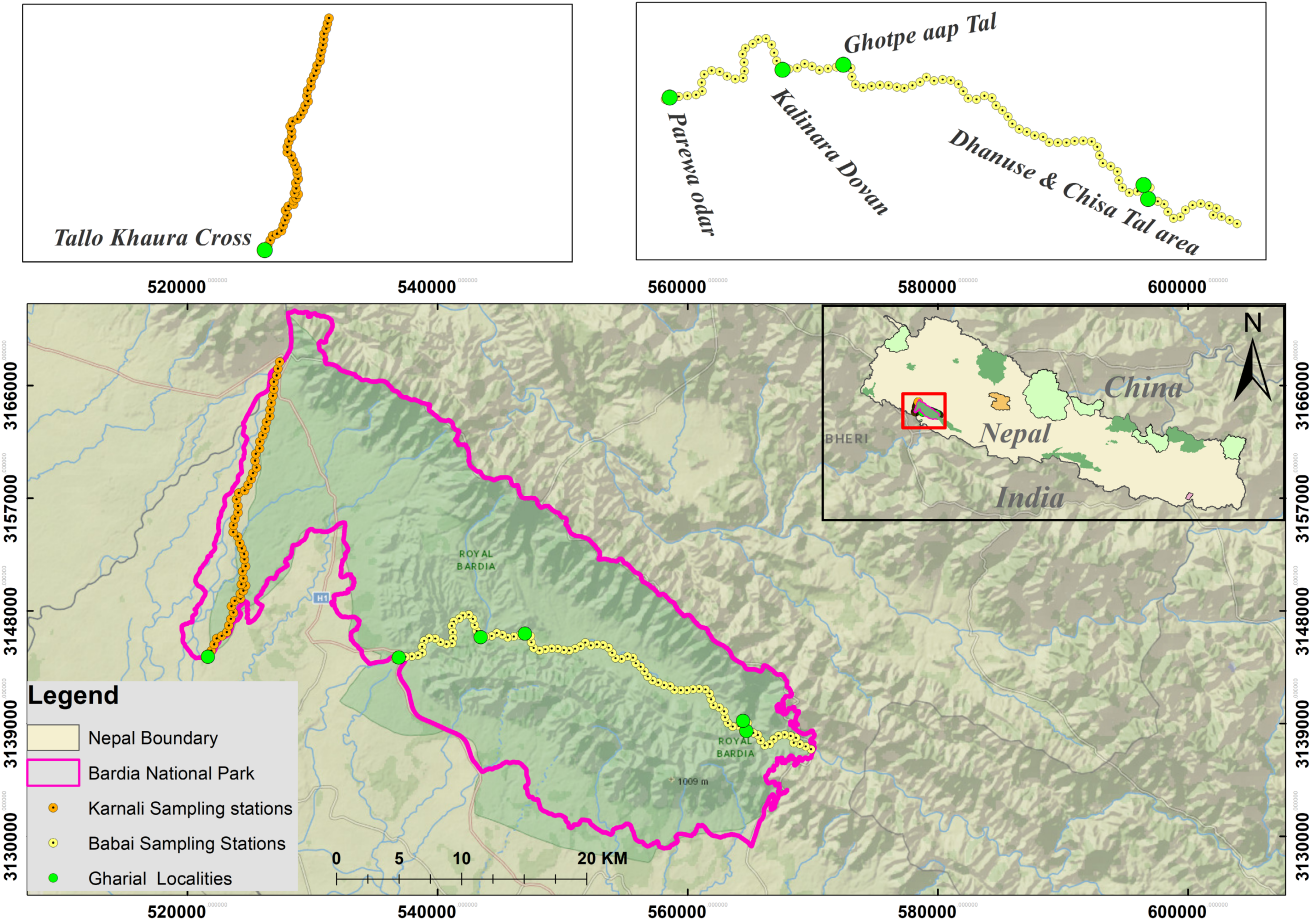
Distribution map of gharial localities inside Bardia National Park is highlighted with light green circles. The sampling stations in Babai River are highlighted with yellow circles, and sampling stations in Karnali River are highlighted with orange circles.

The study was conducted in two river systems (Karnali and Babai) of BNP (28°15′ to 28°35.5′ N and 80°10′ to 81°45′ E) (Figure 2). The Babai is a warm‐water river system that enters BNP from its north‐eastern boundary at Chepang and leaves at Babai Bridge (Parewa Odar), approximately 47 km downstream. The Karnali and Babai rivers, however, are not connected. Karnali is snow‐fed and perennial, originating from the Tibetan Plateau that enters BNP at its north‐western corner at the Chisapani Bridge and bifurcates into two major channels running downstream and south. We studied 30 km downstream that extends from Karnali bridge to Tallo Khaura Cross. The vegetation is sub‐tropical, with floodplain vegetation on river sides, and *Shorea robusta* forest on surrounding uplands. BNP serves as the ecological lifeline for a diverse range of threatened species, including the Asian elephant (*Elephas maximus*), greater one‐horned rhinoceros (*Rhinoceros unicornis*), tiger (*Panthera tigris tigris*), smooth‐coated otter (*Lutra perspicillata*), and mugger crocodile (*Crocodylus palustris*) (Thapa et al., 2018). In this region, over 60% of the population is comprised of Indigenous Tharu communities residing along the rivers. While their primary sources of livelihood are agriculture and animal husbandry, they often face challenges in meeting their daily and incidental needs. Consequently, they also rely on the rivers for resources, including fishing. This additional dependence on river resources can have negative impacts on Gharials and their habitat due to potential conflicts and resource depletion.

### 2.2 Sampling design and data collection

This study was conducted during post‐winter season in Nepal, between February and March 2023. This season is considered as favorable season for Gharial survey and monitoring because of low water level in the river channels compared to other seasons (Nishan et al., 2023). Further, the mild weather during this period allows Gharials to bask on the riverbank for a longer duration, thereby increasing the likelihood of sighting them (Adhikari et al., 2019). The data collection was done in two phases.

#### 2.2.1 Preliminary survey

In February 2023, preliminary surveys were conducted in two phases over a period of 6 days. During the first phase, first author conducted detailed informal interviews with five conservation officials of BNP and two personnel from the National Trust for Nature Conservation (NTNC), who had excellent knowledge of topography, ecological significance, human interactions, conservation efforts, and management considerations necessary for preserving these valuable natural resources of BNP, to understand the potential factors affecting the habitat use of Gharials such as presence of Invasive species and illegal fishing. These interviews assisted us in obtaining an overview of the current Gharial distribution within various segments of these rivers. In the second phase of our study, we used Google Earth Pro (Version: 7.3.4.8642) to digitize the mid‐channel route of the river streams. We focused on the downstream mid‐channel route, covering a total distance of 47 km of the Babai River (Parewa Odar to Chepang) and 30 km of the Karnali River (Karnali bridge to Tallo Khaura Cross), where Gharials have been reported to inhabit. The extracted river channel routes were further divided into sections so that each sub‐sectors can be surveyed in single day to minimize bias in habitat use measures due to the upstream and downstream movement of Gharial individuals within the river (Neupane et al., 2020; Poudyal et al., 2018) (Table 1).

**TABLE 1:** Name and length (km) of section of Babi and Karnali river of Bardia National Park.

River stream	Section	Section length (in km)	Total distance (in km)
Babai river	Parewa Odar – Kalinara	12	47
	Kalinara – Sivapur post	19	
	Sivapur post – Chepang bridge	16	
Karnali river	Karnali bridge – Gaida Machhan	14.5	30
	Gaida Machhan – Tallo Khaura Cross	15.5	

Then, we monitored the Gharial population in the chosen river segments by wooden boat/walking downstream along the riverbanks. This monitoring was repeated three times to observe any differences in the number of Gharial individuals occupying the river segment. Once we confirmed that there were no significant differences in the number of observed individuals during three consecutive surveys (Figure S1), we proceeded with conducting a detailed habitat survey of the Gharial. During the preliminary field visit, an experienced local field guide from BNP, who had prior experience in studying crocodilian species, estimated the slopes of 40 riverbanks. The riverbanks were chosen to represent both sides of the river and varied topographic conditions. Simultaneously, a second experienced local field guide recorded the GPS coordinates of the visually estimated riverbank slopes, considering the absence of Gharials and other megafaunal species to avoid disturbance to species and any potential risks. Next, we validated the numerical values of the virtual slope estimation method using ArcGIS 10.8.1 (ESRI, 2020) by estimating the slope of each recorded point using DEM (Digital Elevation Model, 12.5 m resolution) (ASF, 2021). We observed that the data generated through virtual observation were close to the data generated through the GIS tool. Therefore, we decided to use the virtual estimation method for the major field survey.

#### 2.2.2 Major field survey and recording the habitat characteristics

During March of 2023, a detailed field survey was conducted, which involved generating sampling stations at 500 meter (m) intervals using ArcGIS 10.8.1.(ESRI, 2020). The rationale behind this strategy was that the habitat characteristics would differ significantly at 500 m intervals, and it was less likely that basking Gharials would move across consecutive stations during the survey. Several studies (Bhattarai et al., 2022; Lamichhane et al., 2022; Nishan et al., 2023) have utilized similar methods and assumptions for assessing habitat characteristics of various species of crocodiles, including the Gharials (Neupane et al., 2020). Habitat types were assessed on both sides of the river (left and right bank) at every survey station and at each Gharial sighting location. The survey was conducted during the mid‐day between the hours 8:00–16:00 h as it is assumed that most Gharial individuals would be basking during that time (Adhikari et al., 2019). Further, breeding animals tend to bask in groups during late winter and early spring, and their courtship behavior provides a better opportunity to observe them (Choudhury & Rao, 1982). Additionally, surveys were exclusively conducted during clear sunny weather conditions to mitigate potential biases in certain field measurements, such as water temperature, which were collected on different days.

A team of five individuals including two experienced local field guides aided with binoculars and a range finder moved downstream. The team recorded the number of Gharials and their sex, and age class. The sex was determined by observing distinct morphological feature called “Ghara” present in male adult only. Total length of the Gharial was estimated by calibrating with natural objects or landscape features using binoculars and was assigned into different age classes as hatchlings (<120 cm), juveniles (120–180 cm), sub‐adults (180–270 cm) and adults (>270 cm) (Neupane et al., 2020; Rajbhandari & Acharya, 2015). Based on preliminary field visit and published literature review (Bashyal et al., 2021; Neupane et al., 2020; Poudyal et al., 2018; Rajbhandari & Acharya, 2015), 10 pre‐defined habitat characteristics were identified as potentially influencing Gharial occurrences; and those selected variables included: elevation, river width, mid‐river depth, bank width, river substrate type, river bank slope, water temperature, presence/absence of anthropogenic disturbance, invasive alien plant species and basking sites (see Table 2 for details).

**TABLE 2:** Habitat variables used in the logistic regression analysis with their type, description, and reference.

Variables	Variable category	Type	Description	Reference
Presence or Absence	Ecological variable	Binary	The sampling stations and sighting locations are characterized by the presence or absence of Gharials, where “1” denotes the presence and “0” denotes the absence of Gharials.	
Elevation [Elev]		Continuous	Elevation at mean sea level (m) at each sampling station and sighting location.	Neupane et al. (2020)
Mid‐river depth [MRD]		Continuous	Mid‐river depth (m) at each sampling station and sighting location.	Neupane et al. (2020)
River width [RW]		Continuous	River width (m) at each sampling station and sighting location.	Neupane et al. (2020)
Bank width [BW]		Continuous	Bank width (m) at each sampling station and sighting location.	Neupane et al. (2020)
Riverbank substrate type [RST]		Categorical	Sandy bank, sandy gravel, sandy grassy, sandy, grassy & gravel, gravel, gravel grassy and rocky	Neupane et al. (2020)
Riverbank slope [RBS]		Categorical	Gentle (<15° slope) Moderate (15–25° slope), Moderately steep (25–35° slope), Steep (35–55° slope) & very steep (>55° slope)	He et al. (2020); Nishan et al. (2023); Thong et al. (2018)
Water temperature [WT]		Continuous	Water temperature (°C) at each sampling station and sighting location.	Adhikari et al. (2019); Hussain (1999)
Presence/absence of basking sites [P.A.BS]		Categorical	Present = “1,” absent = “0” of Gharial basking site at sampling station and sighting location.	Bashyal et al. (2021)
Presence/absence of anthropogenic disturbance [P.A.AD]	Disturbance variable	Categorical	Evidence of any anthropogenic disturbance (washing clothes, cattle presence, grass collection, elephant rides, human walking trails, fishing, non‐timber forest products (NTFPs) collection, swimming, gabion wall dikes) present = “1,” absent = “0” at each sampling station and sighting location.	Nishan et al. (2023)
Presence/absence of alien invasive plant species [P.A.Inv]		Categorical	Evidence of any alien invasive plant species present = “1,” absent = “0” at each sampling station and sighting location.	Nishan et al. (2023)

The elevation at each sampling station and sighting location was recorded using a Garmin eTrex 30. River width and Bank width were estimated using the Vortex Optics Razor HD 4000 range finder. The mid‐river depth was estimated using a long‐calibrated bamboo stick which was scaled at every 5 cm interval. River substrate type and presence/absence of [anthropogenic disturbance, invasive alien plant species (such as *Lantana camara, Ageratum houstonianum*, and *Chromolaena odorata*) and basking sites] were virtually estimated (Neupane et al., 2020; Nishan et al., 2023). To avoid the misidentification of the invasive alien species, primary author captured the high‐resolution photograph of the recorded invasive species and conducted a taxonomic validation by cross‐referencing it with the Book “Invasions of Alien Plant Species in Nepal” authored by Shrestha and Shrestha (2021). In our study, we rigorously assessed the presence or absence of Gharial basking sites by comparing riverbank width to the size of adult male Gharials. Sites with riverbanks equal to or larger than the dimensions of adult male Gharials were categorized as “basking site presence,” while those with narrower riverbanks were labeled as “basking site absence.” Further, the water temperature of the river was measured with temperature meter (Hanna HI991300).

### 2.3 Data analysis

The present study utilized a binary logistic regression model to investigate the factors that influence the occurrence of Gharial at a selected site. The response variable was the binary presence or absence of Gharial at both sampling stations and sighting locations, whereas 10 pre‐determined habitat variables (Table 2) were treated as independent (predictor) variables.

The potential issue of multicollinearity was assessed before conducting regression analysis, and the variance inflation factor (VIF) was calculated using the “Faraway” R package (Boomsma, 2014). All independent variables were evaluated for VIF values less than 10, indicating no significant multicollinearity (Bowerman & O'connell, 1990). Next, a global model logistic regression analysis was performed, with all independent variables included under the binomial family and logit link function. In order to rank the potential candidate models, we constructed multiple models and evaluated their performance using Akaike's Information Criterion with correction (AICc) for small sample sizes (Barton & Barton, 2020). The most promising candidate models with delta AIC ≤2 were selected and model averaging (covering 98% weightage (*w)*) was performed to obtain the final models, as suggested by Burnham and Anderson (2001) for minimizing uncertainty.

The “AICmodavg” package Mazerolle and Mazerolle (2017) was employed for this purpose. To assess the predictive performance of the best‐fit model (models with ΔAIC ≤2), we calculated the Area Under Curve (AUC) of the Receiver Operator Characteristics (ROC) using the “ROCR” R package (Sing et al., 2005). The AUC values ranged from 1 to 0. The higher the AUC value, the better the model performance and vice‐versa (Carter et al., 2016). According to Hosmer et al. (2000), receiver operator characteristic (ROC) values ranging from 0.7 to 0.8 are considered to exhibit acceptable discrimination, 0.8 to 0.9 are considered excellent, and values greater than 0.9 are considered superior. A *p*‐value (Pr(>|*z*|)) less than .05 (at a significance level of 5%) was used to determine if the independent variables were significantly associated with the response variable in the model. Further, we utilized G‐test (likelihood ratio test) (Agresti, 2012) to determine if there is a significant relationship between male and female‐biased populations at 5% level of significance. Modeling analysis was conducted using the statistical package: R x 64 4.2.1 (R Core Team, 2020).

## 3 RESULTS

### 3.1 Population abundance and distribution of Gharials in Bardia National Park

**FIGURE 3:**
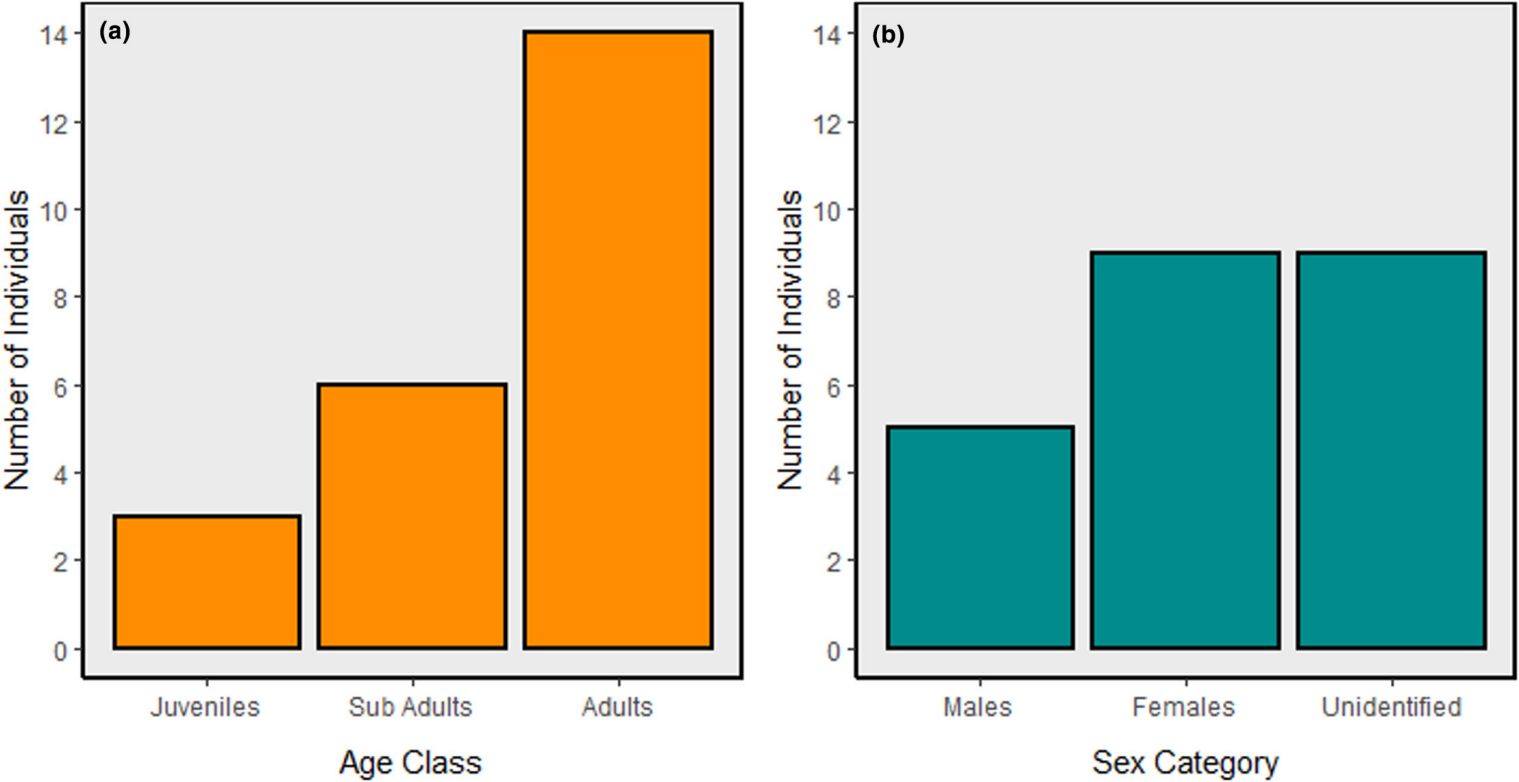
Bar graph showing the distribution of individuals based on age class (subplot a) and sex category (subplot b). Subplot “a” shows the number of juveniles, sub‐adults, and adults, while subplot “b” shows the number of males, females, and unidentified individuals.

A total of 23 individuals of Gharials were recorded during the survey (Figure S1), with 22 individuals sighted in the Babai River and only one in the Karnali River. The total search effort was 1.93 km/h. Among the recorded Gharials, 14 were adults (61%), six were sub‐adults (26%), and three were juveniles (13%). Additionally, the study found that nine individuals could not be identified by sex, while five were identified as male and nine as female (Figure 3). Similarly, 83% of Gharials individuals were found basking, whereas the remainders were found swimming. The sex ratio was found to be 55.56 males per 100 females. However, result did not provide sufficient evidence to support the claim that the Gharial population in BNP has a biased sex ratio (χ^2^ = 1.1429; *p*‐value = .285).

### 3.2 Influencing variables and probability of Gharial occurrence

**FIGURE 4:**
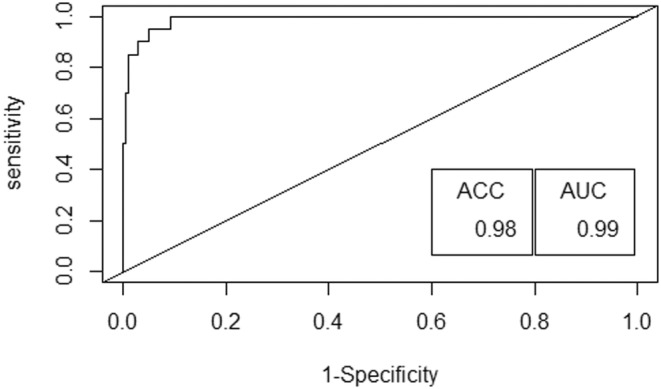
ROC curve for optimal binary logistic regression model, displaying ACC (accuracy) and AUC (area under curve) metrics for performance evaluation.

Among the candidate models generated from binary logistic regression to elucidate the probability of Gharial occurrence, the model with additive effect comprising mid‐river depth, availability of basking site, river width, and water temperature exhibited the lowest Akaike information criterion (AIC) value of 41.70 and the highest weight of 0.15. This model is considered the most parsimonious and provides the best explanation of the probability of Gharial occurrence (Table 3). The dominant model exhibited a remarkable level of proficiency, with an estimated area under the Receiver Operating Characteristic (ROC) curve of 0.99 and an accuracy score of 0.98 (98%) (Figure 4).

**TABLE 3:** Akaike information criterion (AIC) values and weightage of binary logistic regression models, showing the most parsimonious and best‐fitting model for predicting the probability of Gharial occurrence.

Models	df	logLik	AICc	ΔAIC	Weight
Detection ~ (MRD + P.A.BS + RW + WT)	5	−15.70	41.70	0.00	0.15
Detection ~ (MRD + RW + WT)	4	−16.80	41.79	0.09	0.14
Detection ~ (MRD + RST + RW)	9	−11.84	42.61	0.91	0.09
Detection ~ (Elev + MRD + P.A.BS + RW + WT)	6	−15.19	42.80	1.10	0.09
Detection ~ (BW + MRD + RST + RW)	10	−10.86	42.87	1.17	0.08
Detection ~ (Elev + MRD + RW + WT)	5	−16.40	43.10	1.39	0.07
Detection ~ (MRD + P.A.AD + RST + RW)	10	−11.06	43.27	1.57	0.07
Detection ~ (MRD + P.A.AD + RW + WT)	5	−16.50	43.30	1.60	0.07
Detection ~ (MRD + P.A.AD + P.A.BS + RW + WT)	6	−15.52	43.47	1.77	0.06
Detection ~ (MRD + RST + RW + WT)	10	−11.21	43.57	1.87	0.06
Detection ~ (BW + MRD + P.A.BS + RW + WT)	6	−15.59	43.62	1.91	0.06
Detection ~ (MRD + P.A.BS + P.A.Inv + RW + WT)	6	−15.61	43.64	1.94	0.06

Abbreviations: BW, bank width; df, degree of freedom; Elev, elevation; MRD, mid‐river depth; P.A.AD, presence/absence of anthropogenic disturbance; P.A.BS, presence/absence of basking sites; P.A.Inv, presence/absence of invasive species; RST, river substrate type; RW, river width; WT, water temperature.

**FIGURE 5:**
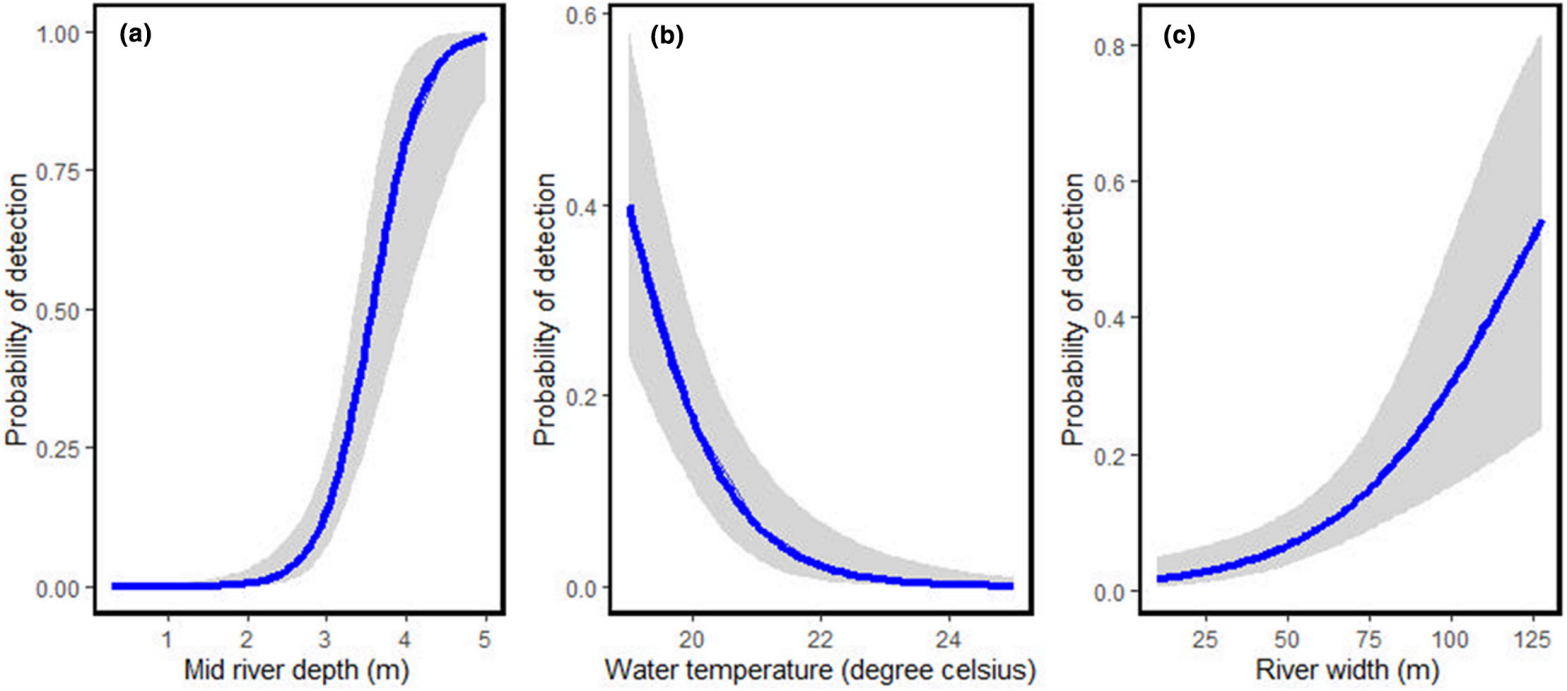
Figure “a,” “b,” and “c” showing the relation of occurrence of detected Gharial with mid‐river depth, water temperature, and river width, respectively.

Out of the 10 habitat predictors investigated in this study, three habitat predictors, mid‐river depth (β = 3.958e+00, SE = 1.370e+00, *p* = .00386), river width (β = 5.598e‐02, SE = 2.481e‐02, *p* = .02404), and water temperature (β = −8.085e‐01, SE = 3.368e‐01, *p* = .01638) were significantly correlated with the probability of Gharial occurrence (Table 4). Results of the model indicate that the probability of detecting Gharials in BNP increases with rise in mid‐river depth, river width, and decreasing water temperature (Table 4 and Figure 5).

**TABLE 4:** Model‐averaged coefficients of predictor variables and their impact on the detection of Gharials in Bardia National Park, with significant variables influencing Gharial habitat use (Pr (> |*z*|) < .05) marked with asterisks (*).

Predictors	Estimate	Std. error	*z* Value	Pr(>|z|)
Intercept	−1.810e+01	1.212e+04	0.001	0.99881
MRD	3.958e+00	1.370e+00	2.889	0.00386**
Factor [P.A.BS]2	1.717e+01	2.521e+03	0.007	0.99457
RW	5.598e‐02	2.481e‐02	2.256	0.02404*
WT	−8.085e‐01	3.368e‐01	2.400	0.01638*
Factor [RST] grassy sandy	2.124e+01	2.175e+04	0.001	0.99922
Factor [RST] grassy, gravel & sandy	3.510e+00	2.602e+04	0.000	0.99989
Factor [RST] gravel	2.484e+00	2.767e+04	0.000	0.99993
Factor [RST] rocky	1.673e+00	2.204e+04	0.000	0.99994
Factor [RST] sandy	2.334e+01	2.175e+04	0.001	0.99914
Factor [RST] sandy gravel	2.019e+01	2.175e+04	0.001	0.99926
Elev	1.235e‐02	1.325e‐02	0.932	0.35138
BW	4.739e‐02	8.060e‐02	0.588	0.55657
Factor [P.A.AD]2	1.358e+00	1.654e+00	0.821	0.41168
Factor [P.A.Inv]2	−1.626e+01	6.625e+03	0.002	0.99804

## 4 DISCUSSION

Given the global decline of Gharial populations caused by anthropogenic disturbance, unique habitat requirements, low hatchling survival rates, and sex‐biased population densities (DNPWC, 2018), urgent large‐scale conservation efforts (eco‐friendly infrastructure development, implementing captive breeding programs, promoting sustainable fisheries management, launching species translocation programs, and collaborative cross‐border monitoring initiative) are necessary for their sustainable conservation. This study identifies the primary distribution locations of Gharials, assesses their current population status, and investigates influential variables affecting their distributions in two major river streams of western Nepal. The findings of this study are profoundly influential, as they provide the essential insights needed to shape infrastructure plans that prioritize environmental conservation, thus promoting robust and informed decision‐making in this critical sphere.

### 4.1 Population abundance of Gharials in Bardia National Park

Government records indicate that since the inception of the Gharial Conservation and Breeding Center (GCBC) in CNP, a total of 1692 captive‐bred Gharial individuals have been successfully released into various river systems throughout Nepal (CNP, 2022). Highest number of individuals were released in Rapti river (972 individuals, 57.45%), followed by Narayani River (419 individuals, 24.76%), Sapta Koshi river (115 individuals, 6.8%), Babai River (110 individuals, 6.5%), Karnali river (41 individuals, 2.42%) and Kali Gandaki River (35 individuals, 2.07%). These releases are an essential component of conservation efforts for the species, aimed at increasing their population and genetic diversity. However, the expected increase in the Gharial population has not been observed (Khadka et al., 2020; Neupane et al., 2020). The lower survival rate of the released individuals highlights the presence of multiple threats to the species, including monsoonal wash‐offs into India, the presence of dams that hinder upstream passage from India to Nepal, natural mortality, and accidental deaths from entanglement in fishing nets (Acharya et al., 2017; Ballouard et al., 2010; Yadav et al., 2022).

The population status and habitat of Gharials in BNP have been the subject of numerous studies (Bashyal et al., 2019, 2021; DNPWC, 2018; Khadka et al., 2008; Thapa et al., 2018), which have provided valuable contributions to conservation efforts aimed at preserving this endangered species. Altogether a total of 23 Gharial individuals (five males, nine females, and nine unidentified) were observed in this study. Similarly, our findings are concurrent to the findings of Bashyal et al. (2021) where they recorded 19 individuals (18 Babai, one Karnali). However, Thapa et al. (2018) reported a relatively higher number of Gharials (*n* = 33) in BNP compared to our study, possibly due to the use of a different survey method, namely the Unmanned Aerial Vehicle (UAV). While majority of Gharial studies (including this) (Bashyal et al., 2021; Chowfin & Leslie, 2014; Neupane et al., 2020; Nishan et al., 2023; Rajbhandari & Acharya, 2015; Singh & Rao, 2017) in multiple sites in India and Nepal employed visual encounter surveys (Crump et al., 1994) while sailing random transects along riverine habitat to estimate the Gharial population size.

The direct count method does not account for imperfect detection (Barão‐Nóbrega et al., 2022). Studies have indicated that imperfect detection is a prevalent issue in crocodilians, despite their substantial size (Balaguera‐Reina et al., 2018; Barão‐Nóbrega et al., 2022). Even Gharials, being among the largest species of crocodilians, may not be exempt from this problem during surveying. Therefore, we recommend combining modern methods such as Acoustic Monitoring, Drone Surveys, Remote Sensing and GIS and Citizen Science with traditional direct count methods to increase the accuracy of Gharial population count. Further, the majority of the Gharials were observed in the Babai River, with only a few individuals sighted in the Karnali river which is consistence with the findings of Bashyal et al. (2021). This may be due to lack of preferred sandbank habitat and high anthropogenic disturbance in Karnali river (Paudel et al., 2015). Further, the diversion of water from the Karnali River into irrigation canals for a commanded area of 38,300 hectares (GoN/DWRI, 2023) is expected to result in a significant reduction in river discharge, which may have adverse effects on the population viability of Gharials inhabiting the Karnali River. Therefore, this anthropogenic water extraction for irrigation purposes requires careful consideration to ensure the conservation of the species and the maintenance of the ecological integrity of the river ecosystem. In addition, Vashistha et al. (2021) observed 72 individuals from the Katerniaghat Wildlife Sanctuary (KWS), which is located downstream of the Karnali river. It is possible that KWS Gharial population might have been formed by individuals released in Nepal who moved downstream (particularly hatchlings and juveniles) during periods of high‐water levels or flood, but movement data to support this supposition is lacking. The Girijapuri barrage, located within the KWS is equipped with control gates that allow water to flow downstream but restrict the movement of Gharials upstream, once they cross the Girijapuri barrage gates and enter the Ghaghara River (Vashistha et al., 2021). Furthermore, evidence of breeding population of Gharial in Gaire khola – contributing river tributaries to Mohana River (5 km aerial stretch from western channel of Karnali River) – shows potential for possible movement of reptiles especially in high floods and their breeding success in an areas due low level of threats (especially illegal fishing and sand and gravel extraction) and availability of good nesting habitat.

The demographic composition of a species is a crucial element in population dynamics studies, as it can provide valuable insight into population health and reproductive success (di Sciara, 2018; Hoyle & Maunder, 2004). In our investigation, most Gharials observed were adults, while the number of sub‐adults and juveniles was relatively small. These findings imply that recruitment levels within the population may not be sufficient to maintain a healthy population size. Moreover, the high proportion of Gharials with undetermined sex (39%), as observed in our study, raises significant concerns as it limits our ability to comprehend the population dynamics and the reproductive potential of the species. Similar findings were also reported by other studies that recorded a relatively higher number of unidentified sex Gharials (Bashyal et al., 2021; Khadka, 2011; Neupane et al., 2020; Poudyal et al., 2018). Use of genetic markers for sex identification is advisable yet multiple‐year survey using current protocol is recommended.

A majority (83%) of the Gharials were found basking on sandy riverbanks, which is consistent with the findings of previous studies conducted by Neupane et al. (2020), Rajbhandari and Acharya (2013), Hussain (2009), and Nair (2010). The underlying reason for the observed preference for sandy riverbanks among Gharials may be attributed to their greater ease in crawling on sandy surfaces, in contrast to rocky or clay ones (Neupane et al., 2020). The sand found on riverbanks with lower moisture content acts as a moderator of extreme hot or cold environmental conditions, which ultimately lowers the likelihood of desiccation while basking in sun (Hussain, 2009; Neupane et al., 2020). Given the affinity of Gharials for sandy riverbanks as basking sites, it is advisable to prioritize the preservation and protection of such habitats in conservation initiatives. This may necessitate measures such as managing water flow and preventing soil erosion to ensure the continued availability of suitable basking areas.

### 4.2 Influencing variables and probability of Gharial occurrence

The results of our binary logistic regression analysis indicate that three habitat predictors, namely mid‐river depth, river width, and water temperature, were significantly associated with the likelihood of Gharial occurrence in BNP. However, the remaining variables did not demonstrate any significant predictive power in our analysis.

Our study's findings, which demonstrate a positive correlation between mid‐river depth and the likelihood of Gharial occurrence, are consistent with earlier research (Hussain, 2009; Neupane et al., 2020; Rajbhandari & Acharya, 2015; Yadav et al., 2022) that highlight deep water areas as favorable habitats for Gharials. The availability of escape cover, such as deep water near basking sites, is a critical factor in their habitat selection since it allows them to retreat into water for safety when they feel threatened or disturbed by human activity (Hussain, 2009; Neupane et al., 2020). Furthermore, female Gharials often choose nesting sites in areas near deeper water channels, which provide improved access to prey (Gupta et al., 2012), suggesting that the relationship between mid‐river depth and Gharial occurrence may also be linked to reproductive success. Significant population recovery, with evidence of breeding success, has been recorded through the strategic selection of sites with deep‐water channels along the main tributaries of the Karnali River‐Geruwa, flowing through protected areas, in the recent past (Personal communication: Bishnu Shrestha, Former Chief Warden‐BNP). With the exception of the Babai River, the environmental flow of water discharge in the Karnali River is worth considering, given the changes in the level of water flow in the channel due to the construction or diversion of water in RJKIS, particularly during the dry season. Further, it is plausible that Gharials' preference for deep water segments is linked to their thermoregulation needs. Deeper water segments maintain warmer temperatures below the surface during winter and cooler temperatures in summer, providing a temperature gradient that Gharials can utilize for efficient temperature regulation. However, this hypothesis requires further investigation to establish its validity.

There exists a significant positive correlation between river width and Gharial occurrence, providing evidence that wider rivers may offer more suitable habitats for these reptiles. One possible explanation for this relationship is that wider rivers provide more space, diverse microhabitats, and better connectivity between different parts of the river ecosystem, which are important factors for Gharial survival and reproduction. It is important to note that our results differ from those of previous studies conducted by Neupane et al. (2020) and Yadav et al. (2022), which found no significant impact of river width on the probability of Gharial occurrence. Further research is needed to reconcile these inconsistencies and gain a more comprehensive understanding of the ecological requirements and behaviors of Gharials. Further, due to the implementation of BBDMP, there will be a substantial increase in the flow of water in the Babai River (GoN, 2023), leading to alterations in the river's width. This change in flow may result in widening of the river in certain locations, while in other areas, it could cause erosion and lead to the narrowing of the river. This has both positive (more suitable habitat for feeding) and negative (reduce the availability of suitable nesting sites) implications for the conservation of the Gharial (Vashistha et al., 2021). Future studies should assess altered flow regime of Babai River due to the BBDMP and its impact on Gharial feeding and nesting ecology.

Our study revealed a significant negative correlation between water temperature and the probability of Gharial occurrence, which supports the findings of previous studies conducted by Shrestha (2000), and Adhikari et al. (2019). In winter, with air temperatures at around 18°C (64°F) and water temperatures near 17°C (63°F), Gharials basked throughout the day. Conversely, in summer, when air temperatures soared to about 35°C (95°F) and water temperatures reached approximately 25°C (77°F), gharials basked less and avoided midday sunbathing (Shrestha, 2000). Similarly, Adhikari et al. (2019) studied the effect of temperature fluctuation on the thermoregulatory behavior of captive Gharials and found that Gharials tended to remain in pools in the morning when the water temperature was warmer than the air temperature. However, more Gharials tended to move outside the water for basking when the water temperature dropped relative to the air temperature. These findings hold profound implications for the comprehensive conservation and sustainable management of Gharial populations in the wild. To bolster these initiatives, it is imperative to prioritize the development of environmentally conscious infrastructure that not only addresses economic demands but also harmonizes with the overarching objectives of conservation. This mandates a holistic strategy encompassing the preservation of critical habitats and meticulous consideration of the myriad factors influencing Gharial ecology, encompassing aspects like basking patterns, feeding behaviors, and nesting preferences. By highlighting the link between water temperature and Gharial behavior, our study also suggests that Gharials are sensitive to environmental conditions, which could make them particularly vulnerable to the effects of climate change.

## 5 CONCLUSION

This study contributes to the selection of interventions aimed at protecting Gharials and their associated habitats, specifically sandy banks in BNP. The Gharial population is comparable to previous studies and has remained stable over the years. This study establishes a population baseline for Gharials within the river system before the construction of large infrastructure projects, such as dams and irrigation canals. Continuous monitoring of Gharial populations along the river system after water release and/or diversion is recommended. This will help to evaluate the impact of large infrastructure projects, specifically the BBDMP and RJKIS, on the population and their associated habitat characteristics. Both projects are scheduled to be completed around 2025 or beyond.

The results of the best fit model indicate that the likelihood of detecting Gharials in BNP increases with higher mid‐river depth and river width, as well as lower water temperature. These factors are crucial for developing sustainable infrastructure plans that ensure species conservation and maintain the ecological integrity of the river ecosystem. Considering the high demand for large infrastructure construction, such as dams in the river channel for electricity and irrigation, the assessment of environmental flow is crucial and likely to benefit Gharials.

Within BNP, riverbanks are protected yet habitat outside the core areas suffers from ongoing habitat degradation such as gravel extraction and mining. Use of citizen scientists (Bonney et al., 2014) to collect unbiased data to monitor both the population and habitat is recommended. There are analytical tools for robust analysis of unbiased data collected from the citizen scientist.

## AUTHOR CONTRIBUTIONS

**Bijaya Dhami:** Conceptualization (lead); data curation (lead); formal analysis (lead); funding acquisition (lead); investigation (lead); methodology (lead); project administration (lead); resources (lead); supervision (lead); writing – original draft (lead); writing – review and editing (equal). **Tek Maraseni:** Conceptualization (supporting); resources (equal); supervision (equal); validation (equal); writing – review and editing (equal). **Kanchan Thapa:** Conceptualization (equal); supervision (equal); writing – review and editing (equal). **Nishan K. C.:** Investigation (equal); resources (equal); writing – review and editing (equal). **Sanskar Subedi:** Investigation (equal); resources (equal); writing – review and editing (equal). **Shreejan Gautam:** Investigation (equal); resources (equal); writing – review and editing (equal). **Santosh Ayer:** Investigation (equal); resources (equal); writing – review and editing (equal). **Erin Bayne:** Conceptualization (equal); investigation (equal); writing – review and editing (equal).

## Supporting information


Figure S1

## Data Availability

Link to the data can be accessed at zenedo.org through https://doi.org/10.5281/zenodo.813498.

## References

[ece310661-bib-0001] Abell, R. , Lehner, B. , Thieme, M. , & Linke, S. (2017). Looking beyond the fenceline: Assessing protection gaps for the world's rivers. Conservation Letters, 10, 384–394.

[ece310661-bib-0002] Acharya, K. P. , Khadka, B. K. , Jnawali, S. R. , Malla, S. , Bhattarai, S. , Wikramanayake, E. , & Köhl, M. (2017). Conservation and population recovery of gharials (*Gavialis gangeticus*) in Nepal. Herpetologica, 73, 129–135.

[ece310661-bib-0003] Adhikari, S. , Poudel, D. , & Bolakhe, S. (2019). Effect of temperature fluctuation on thermoregulatory behavior of gharial in winter season (a case study of gharial conservation breeding Centre, Chitwan National Park, Nepal). Research in Zoology, 9, 1–6.

[ece310661-bib-0004] Agresti, A. (2012). Categorical data analysis. John Wiley & Sons.

[ece310661-bib-0006] ASF . (2021). Alaska satellite facility. Alos Palsar . NASA.

[ece310661-bib-0007] Balaguera‐Reina, S. A. , Venegas‐Anaya, M. D. , Rivera‐Rivera, B. , Morales Ramírez, D. A. , & Densmore, L. D., III . (2018). How to estimate population size in crocodylians? Population ecology of American crocodiles in Coiba Island as study case. Ecosphere, 9, e02474.

[ece310661-bib-0008] Balian, E. V. , Segers, H. , Martens, K. , & Lévéque, C. (2008). The freshwater animal diversity assessment: An overview of the results. Springer.

[ece310661-bib-0009] Ballouard, J.‐M. , Priol, P. , Oison, J. , Ciliberti, A. , & Cadi, A. (2010). Does reintroduction stabilize the population of the critically endangered gharial (*Gavialis gangeticus*, Gavialidae) in Chitwan National Park, Nepal? Aquatic Conservation: Marine and Freshwater Ecosystems, 20, 756–761.

[ece310661-bib-0010] Barão‐Nóbrega, J. A. L. , González‐Jaurégui, M. , & Jehle, R. (2022). N‐mixture models provide informative crocodile (*Crocodylus moreletii*) abundance estimates in dynamic environments. PeerJ, 10, e12906. 35341055 10.7717/peerj.12906 PMC8944345

[ece310661-bib-0011] Barton, K. , & Barton, M. K. (2020). *MuMIn: multi‐model inference. R package version 1.43.17. Version 1*. p. 18.

[ece310661-bib-0012] Bashyal, A. , Gumbs, R. , Bhandari, A. , & Khadka, B. (2019). Confirmed record of gharial (*Gavialis gangeticus*) nests and hatchlings in the Babai River, Bardia National Park, Nepal. Crocodile Specialist Group Newsletter, 38, 10–11.

[ece310661-bib-0013] Bashyal, A. , Shrestha, S. , Luitel, K. P. , Yadav, B. P. , Khadka, B. , Lang, J. W. , & Densmore, L. D. (2021). Gharials (*Gavialis gangeticus*) in Bardiya National Park, Nepal: Population, habitat and threats. Aquatic Conservation: Marine and Freshwater Ecosystems, 31, 2594–2602. 10.1002/aqc.3649

[ece310661-bib-0014] Bhattarai, D. , Lamichhane, S. , Pandeya, P. , Bhattarai, S. , Gautam, J. , Kandel, R. C. , & Pokheral, C. P. (2022). Status, distribution and habitat use by mugger crocodile (*Crocodylus palustris*) in and around Koshi Tappu wildlife reserve, Nepal. Heliyon, 8, e10235. 10.1016/j.heliyon.2022.e10235 36061015 PMC9434049

[ece310661-bib-0015] Bonney, R. , Shirk, J. L. , Phillips, T. B. , Wiggins, A. , Ballard, H. L. , Miller‐Rushing, A. J. , & Parrish, J. K. (2014). Next steps for citizen science. Science, 343(6178), 1436–1437. 24675940 10.1126/science.1251554

[ece310661-bib-0016] Boomsma, A. (2014). Regression diagnostics with R. Department of Statistics & Measurement Theory, University of Groningen.

[ece310661-bib-0017] Bowerman, B. L. , & O'Connell, R. T. (1990). Linear statistical models: An applied approach. Brooks/Cole.

[ece310661-bib-0018] Braulik, G. T. , Arshad, M. , Noureen, U. , & Northridge, S. P. (2014). Habitat fragmentation and species extirpation in freshwater ecosystems; causes of range decline of the Indus River dolphin (*Platanista gangetica* minor). PLoS One, 9, e101657. 25029270 10.1371/journal.pone.0101657 PMC4100755

[ece310661-bib-0019] Burnham, K. P. , & Anderson, D. R. (2001). Kullback‐Leibler information as a basis for strong inference in ecological studies. Wildlife Research, 28, 111–119.

[ece310661-bib-0020] Carter, J. V. , Pan, J. , Rai, S. N. , & Galandiuk, S. (2016). ROC‐ing along: Evaluation and interpretation of receiver operating characteristic curves. Surgery, 159, 1638–1645. 26962006 10.1016/j.surg.2015.12.029

[ece310661-bib-0021] Choudhury, B. C. , & Rao, R. K. (1982). Status survey of crocodile populations. A field guide. Central Crocodile Breeding and Management Training Institute.

[ece310661-bib-0022] Chowfin, S. M. , & Leslie, A. J. (2014). A multi‐method approach for the inventory of the adult population of a critically endangered crocodilian, the gharial (*Gavialis gangeticus*) at Dhikala, Corbett Tiger Reserve incorporating direct counts and trail cameras. International Journal of Biodiversity and Conservation, 6, 148–158.

[ece310661-bib-0023] CITES . (2021). Index of CITES species (pp. 1–5). CITES Secretariat; UNEP‐WCMC.

[ece310661-bib-0024] CNP . (2022). Chitwan National Park: Annual report (2022). Department of National Parks and Wildlife Conservation, Chitwan National Park (CNP).

[ece310661-bib-0025] Collen, B. , Whitton, F. , Dyer, E. E. , Baillie, J. E. M. , Cumberlidge, N. , Darwall, W. R. T. , Pollock, C. , Richman, N. I. , Soulsby, A. , & Böhm, M. (2014). Global patterns of freshwater species diversity, threat and endemism. Global Ecology and Biogeography, 23, 40–51. 26430385 10.1111/geb.12096 PMC4579866

[ece310661-bib-0026] Crump, M. L. , Scott, N. J., Jr. , Heyer, W. E. , Donelly, M. A. , McDiarmid, R. W. , Hayek, L. A. C. , & Foster, M. S. (1994). Visual encounter surveys. In Measuring and monitoring biological diversity: Standard methods for amphibians. Smithsonian Institution Press.

[ece310661-bib-0027] Darwall, W. , Bremerich, V. , De Wever, A. , Dell, A. I. , Freyhof, J. , Gessner, M. O. , Grossart, H. , Harrison, I. , Irvine, K. , & Jähnig, S. C. (2018). The Alliance for freshwater life: A global call to unite efforts for freshwater biodiversity science and conservation. Aquatic Conservation: Marine and Freshwater Ecosystems, 28, 1015–1022.

[ece310661-bib-0028] Das, D. K. (2006). Environmental impact of inter‐basin water transfer projects: Some evidence from Canada. Economic and Political Weekly, 41, 1703–1707.

[ece310661-bib-0029] Devkota, R. , Bhattarai, U. , Devkota, L. , & Maraseni, T. N. (2020). Assessing the past and adapting to future floods: A hydro‐social analysis. Climatic Change, 163(2), 1065–1082.

[ece310661-bib-0030] Dhami, B. , Neupane, B. , Devkota, B. P. , Maraseni, T. , Sadadev, B. M. , Bista, S. , Adhikari, A. , Chhetri, N. B. , Panta, M. , & Stewart, A. B. (2023). Factors affecting the occupancy of Chinese pangolins (*Manis pentadactyla*) suggest a highly specialized ecological niche. Ecosphere, 14, e4356.

[ece310661-bib-0031] di Sciara, G. N. (2018). Encyclopedia of marine mammals. Aquatic Mammals, 44, 595–596.

[ece310661-bib-0032] DNPWC . (2018). Gharial conservation action plan for Nepal (2018–2022) (p. 18). Department of National Parks and Wildlife Conservation, Ministry of Forests and Environment.

[ece310661-bib-0033] Dudgeon, D. (2000). Large‐scale hydrological changes in tropical Asia: Prospects for riverine biodiversity: The construction of large dams will have an impact on the biodiversity of tropical Asian rivers and their associated wetlands. Bioscience, 50, 793–806.

[ece310661-bib-0034] Dudgeon, D. , Arthington, A. H. , Gessner, M. O. , Kawabata, Z.‐I. , Knowler, D. J. , Lévêque, C. , Naiman, R. J. , Prieur‐Richard, A.‐H. , Soto, D. , & Stiassny, M. L. J. (2006). Freshwater biodiversity: Importance, threats, status and conservation challenges. Biological Reviews, 81, 163–182. 16336747 10.1017/S1464793105006950

[ece310661-bib-0035] ESRI . (2020). ArcGIS Desktop: Release 10.8.1. Environmental Systems Research Institute.

[ece310661-bib-0037] GoN . (1973). National parks and wildlife conservation act . Government of Nepal, Nepal Law Commission, Nepal.

[ece310661-bib-0038] GoN . (2023). Bheri Babai diversion multipurpose project (BBDMP) . Ministry of Energy, Water Resources and Irrigation. https://www.bbdmp.gov.np/

[ece310661-bib-0039] GoN/DWRI . (2023). Rani Jamara Kulariya irrigation project Tikapur, Kailali . Goverment of Nepal Ministry Energy, Water Resources and Irrigation, Department of Water Resources and Irrigation. https://rjkip.gov.np/

[ece310661-bib-0040] Gumbs, R. , Gray, C. L. , Wearn, O. R. , & Owen, N. R. (2018). Tetrapods on the EDGE: Overcoming data limitations to identify phylogenetic conservation priorities. PLoS One, 13, e0194680. 29641585 10.1371/journal.pone.0194680 PMC5894989

[ece310661-bib-0041] Gupta, B. K. , Sarkar, U. K. , & Bhardwaj, S. K. (2012). Assessment of habitat quality with relation to fish assemblages in an impacted river of the Ganges basin, northern India. Environmentalist, 32, 35–47.

[ece310661-bib-0042] Harrison, I. , Abell, R. , Darwall, W. , Thieme, M. L. , Tickner, D. , & Timboe, I. (2018). The freshwater biodiversity crisis. Science, 362, 1369. 10.1126/science.aav9242 30573621

[ece310661-bib-0043] He, F. , Bremerich, V. , Zarfl, C. , Geldmann, J. , Langhans, S. D. , David, J. N. W. , Darwall, W. , Tockner, K. , & Jähnig, S. C. (2018). Freshwater megafauna diversity: Patterns, status and threats. Diversity and Distributions, 24, 1395–1404.

[ece310661-bib-0044] He, F. , Zarfl, C. , Bremerich, V. , David, J. N. W. , Hogan, Z. , Kalinkat, G. , Tockner, K. , & Jähnig, S. C. (2019). The global decline of freshwater megafauna. Global Change Biology, 25, 3883–3892. 10.1111/gcb.14753 31393076

[ece310661-bib-0045] He, F. , Zarfl, C. , Bremerich, V. , Henshaw, A. , Darwall, W. , Tockner, K. , & Jaehnig, S. C. (2017). Disappearing giants: A review of threats to freshwater megafauna. Wiley Interdisciplinary Reviews Water, 4, e1208.

[ece310661-bib-0046] He, W. , Ye, C. , Sun, J. , Xiong, J. , Wang, J. , & Zhou, T. (2020). Dynamics and drivers of the alpine timberline on Gongga mountain of Tibetan plateau‐adopted from the Otsu method on google earth engine. Remote Sensing, 12, 2651.

[ece310661-bib-0047] Hosmer, D. W. , Lemeshow, S. , & Lemeshow, S. (2000). Applied logistic regression: Wiley series in probability and statistics: Texts and References section. Wiley.

[ece310661-bib-0048] Hoyle, S. D. , & Maunder, M. N. (2004). A Bayesian integrated population dynamics model to analyze data for protected species. Animal Biodiversity and Conservation, 27, 247–266.

[ece310661-bib-0049] Hussain, S. A. (1999). Reproductive success, hatchling survival and rate of increase of gharial *Gavialis gangeticus* in National Chambal Sanctuary, India. Biological Conservation, 87, 261–268.

[ece310661-bib-0050] Hussain, S. A. (2009). Basking site and water depth selection by gharial *Gavialis gangeticus* Gmelin 1789 (Crocodylia, Reptilia) in National Chambal Sanctuary, India and its implication for river conservation. Aquatic Conservation: Marine and Freshwater Ecosystems, 19, 127–133.

[ece310661-bib-0051] Jeliazkov, A. , Gavish, Y. , Marsh, C. J. , Geschke, J. , Brummitt, N. , Rocchini, D. , Haase, P. , Kunin, W. E. , & Henle, K. (2022). Sampling and modelling rare species: Conceptual guidelines for the neglected majority. Global Change Biology, 28, 3754–3777. 35098624 10.1111/gcb.16114

[ece310661-bib-0052] Khadka, B. (2011). Gharial and mugger monitoring in the Narayani and Rapti rivers of Chitwan National Park. Crocodile Specialist Group Newsletter, 30, 11–14.

[ece310661-bib-0053] Khadka, B. , Bashyal, A. , Luitel, K. P. , & Kandel, R. C. (2020). Nesting ecology of gharials (*Gavialis gangeticus*): Implications from in situ and ex situ conservation programs in Chitwan National Park, Nepal. Herpetologica, 76, 297–303.

[ece310661-bib-0054] Khadka, M. , Kafley, H. , & Thapaliya, B. P. (2008). Population status and distribution of gharial (*Gavialis gangeticus*) in Nepal. In *Forum of natural resource managers report, Nepal*.

[ece310661-bib-0055] Lakra, W. S. , Sarkar, U. K. , Dubey, V. K. , Sani, R. , & Pandey, A. (2011). River inter linking in India: Status, issues, prospects and implications on aquatic ecosystems and freshwater fish diversity. Reviews in Fish Biology and Fisheries, 21, 463–479.

[ece310661-bib-0056] Lamichhane, S. , Bhattarai, D. , Karki, J. B. , Gautam, A. P. , Pandeya, P. , Tirpathi, S. , & Mahat, N. (2022). Population status, habitat occupancy and conservation threats to mugger crocodile (*Crocodylus palustris*) in Ghodaghodi lake complex, Nepal. Global Ecology and Conservation, 33, e01977. 10.1016/j.gecco.2021.e01977

[ece310661-bib-0057] Lang, J. , Chowfin, S. , & Ross, J. P. (2019). Gavialis gangeticus. IUCN Red List of Threatened Species, 8235, 30.

[ece310661-bib-0058] Laurance, W. F. , Peletier‐Jellema, A. , Geenen, B. , Koster, H. , Verweij, P. , Van Dijck, P. , Lovejoy, T. E. , Schleicher, J. , & Van Kuijk, M. (2015). Reducing the global environmental impacts of rapid infrastructure expansion. Current Biology, 25, R259–R262. 25754645 10.1016/j.cub.2015.02.050

[ece310661-bib-0059] Maskey, T. M. (1989). Movement and survival of captive‐reared gharial Gavialis gangeticus in the Narayani River, Nepal . (PhD Thesis). University of Florida.

[ece310661-bib-0060] Mazerolle, M. J. , & Mazerolle, M. M. J. (2017). *Package ‘AICcmodavg’*. Model selection and multimodel inference based on (Q)AIC(c). R Package, 281.

[ece310661-bib-0061] Nair, T. (2010). Ecological and anthropogenic covariates influencing gharial Gavialis gangeticus distribution and habitat use in Chambal River, India . Unpublished Master's Thesis.

[ece310661-bib-0062] Neupane, B. , Singh, B. K. , Poudel, P. , Panthi, S. , & Khatri, N. D. (2020). Habitat occupancy and threat assessment of gharial (*Gavialis gangeticus*) in the Rapti River, Nepal. Global Ecology and Conservation, 24, e01270. 10.1016/j.gecco.2020.e01270

[ece310661-bib-0063] Nilsson, C. , & Berggren, K. (2000). Alterations of riparian ecosystems caused by river regulation: Dam operations have caused global‐scale ecological changes in riparian ecosystems. how to protect river environments and human needs of rivers remains one of the most important questions of our time. Bioscience, 50, 783–792.

[ece310661-bib-0064] Nilsson, C. , Reidy, C. A. , Dynesius, M. , & Revenga, C. (2005). Fragmentation and flow regulation of the world's large river systems. Science, 308, 405–408. 15831757 10.1126/science.1107887

[ece310661-bib-0065] Nishan, K. C. , Neupane, B. , Belbase, B. , Dhami, B. , Bist, B. S. , Basyal, C. R. , & Bhattarai, S. (2023). Factors influencing the habitat selection of mugger crocodile (*Crocodylus palustris*) and its conservation threats in the Rapti River of Chitwan National Park, Nepal. Global Ecology and Conservation, 42, e02406. 10.1016/j.gecco.2023.e02406

[ece310661-bib-0066] Pant, G. , Maraseni, T. , Apan, A. , & Allen, B. L. (2021). Predicted declines in suitable habitat for greater one‐horned rhinoceros (*Rhinoceros unicornis*) under future climate and land use change scenarios. Ecology and Evolution, 11, 18288–18304. 35003673 10.1002/ece3.8421 PMC8717310

[ece310661-bib-0067] Paudel, S. , Timilsina, Y. P. , Lewis, J. , Ingersoll, T. , & Jnawali, S. R. (2015). Population status and habitat occupancy of endangered river dolphins in the Karnali River system of Nepal during low water season. Marine Mammal Science, 31, 707–719.

[ece310661-bib-0068] Pelicice, F. M. , Pompeu, P. S. , & Agostinho, A. A. (2015). Large reservoirs as ecological barriers to downstream movements of Neotropical migratory fish. Fish and Fisheries, 16, 697–715.

[ece310661-bib-0069] Poudyal, L. P. , Dahal, B. , Lamichhane, B. R. , & Shrestha, P. (2018). Population status and distribution of gharial in Rivers of Chitwan National Park. Government of Nepal, Ministry of Forests and Environment.

[ece310661-bib-0070] R Core Team . (2020). R: A language and environment for statistical computing. R Foundation for Statistical Computing.

[ece310661-bib-0071] Rajbhandari, S. L. , & Acharya, P. M. (2013). Population, basking and hatching success of Gavialis gangeticus in Narayani river, Chitwan National Park, Nepal. Journal of Natural History Museum, 27, 1–11.

[ece310661-bib-0072] Rajbhandari, S. L. , & Acharya, P. M. (2015). Study of investigation of population, habitat and hatching success of Gavialis gangeticus in Narayani River of Chitwan National Park. Rufford Small Grant Found, UK.

[ece310661-bib-0073] Reid, A. J. , Carlson, A. K. , Creed, I. F. , Eliason, E. J. , Gell, P. A. , Johnson, P. T. J. , Kidd, K. A. , MacCormack, T. J. , Olden, J. D. , & Ormerod, S. J. (2019). Emerging threats and persistent conservation challenges for freshwater biodiversity. Biological Reviews, 94, 849–873. 30467930 10.1111/brv.12480

[ece310661-bib-0074] Renöfält, B. M. , Jansson, R. , & Nilsson, C. (2010). Effects of hydropower generation and opportunities for environmental flow management in Swedish riverine ecosystems. Freshwater Biology, 55, 49–67.

[ece310661-bib-0076] Shrestha, B. B. , & Shrestha, K. K. (2021). Invasions of alien plant species in Nepal: Patterns and process. In T. Pullaiah & M. R. Ielmini (Eds.), Invasive alien species: Observations and issues from around the world (Vol. 2, pp. 168–183). Wiley.

[ece310661-bib-0077] Shrestha, T. K. (2000). Herpetology of Nepal: A field guide to amphibians and reptiles of trans‐Himalayan region of Asia. Steven Simpson Books.

[ece310661-bib-0078] Simkin, R. D. , Seto, K. C. , McDonald, R. I. , & Jetz, W. (2022). Biodiversity impacts and conservation implications of urban land expansion projected to 2050. Proceedings of the National Academy of Sciences of the United States of America, 119, e2117297119. 35286193 10.1073/pnas.2117297119 PMC8944667

[ece310661-bib-0079] Sing, T. , Sander, O. , Beerenwinkel, N. , & Lengauer, T. (2005). ROCR: Visualizing classifier performance in R. Bioinformatics, 21, 3940–3941. 16096348 10.1093/bioinformatics/bti623

[ece310661-bib-0080] Singh, H. , & Rao, R. J. (2017). Status, threats and conservation challenges to key aquatic fauna (crocodile and dolphin) in National Chambal Sanctuary, India. Aquatic Ecosystem Health & Management, 20, 59–70.

[ece310661-bib-0081] Sutton, L. J. , Ibañez, J. C. , Salvador, D. I. , Taraya, R. L. , Opiso, G. S. , Senarillos, T. L. P. , & McClure, C. J. W. (2023). Priority conservation areas and a global population estimate for the critically endangered Philippine eagle. Animal Conservation. 10.1111/acv.12854

[ece310661-bib-0082] Thapa, G. J. , Thapa, K. , Thapa, R. , Jnawali, S. R. , Wich, S. A. , Poudyal, L. P. , & Karki, S. (2018). Counting crocodiles from the sky: Monitoring the critically endangered gharial (*Gavialis gangeticus*) population with an unmanned aerial vehicle (UAV). Journal of Unmanned Vehicle Systems, 6, 71–82. 10.1139/juvs-2017-0026

[ece310661-bib-0083] Thong, P. , Pebam, R. , & Sahoo, U. K. (2018). A geospatial approach to understand the dynamics of shifting cultivation in Champhai district of Mizoram, north‐East India. Journal of the Indian Society of Remote Sensing, 46, 1713–1723.

[ece310661-bib-0084] Vashistha, G. , Mungi, N. A. , Lang, J. W. , Ranjan, V. , Dhakate, P. M. , Khudsar, F. A. , & Kothamasi, D. (2021). Gharial nesting in a reservoir is limited by reduced river flow and by increased bank vegetation. Scientific Reports, 11, 1–12. 10.1038/s41598-021-84143-7 33637782 PMC7910305

[ece310661-bib-0085] Whitaker, R. , Rajamani, V. , Basu, D. , & Balakrishnan, V. (1974). Preliminary survey of the gharial, Gavialis gangeticus . Madras Snake Park Trust Report. 16.

[ece310661-bib-0086] WWF . (2016). Living planet report 2016. Risk and resilience in a new era. WWF.

[ece310661-bib-0087] Yadav, R. K. , Lamichhane, S. , Thanet, D. R. , Rayamajhi, T. , Bhattarai, S. , Bashyal, A. , & Lamichhane, B. R. (2022). Gharial (*Gavialis gangeticus*, Gmelin, 1789) abundance in the Rapti River, Chitwan National Park, Nepal. Ecology and Evolution, 12, 1–11. 10.1002/ece3.9425 PMC9579734 36267686

