# Supplementary material for: Gharial (Gavialis gangeticus) conservation in Bardia National Park, Nepal: Assessing population structure and habitat characteristics along the river channel amidst infrastructure development
Source: Ecol Evol. 2023 Nov 7;13(11):e10661. doi: 10.1002/ece3.10661 (PMC10630156; doi:10.1002/ece3.10661)
Supplement: Supplementary file 1 — Figure S1 [file ECE3-13-e10661-s001.docx]

**Supporting Document**


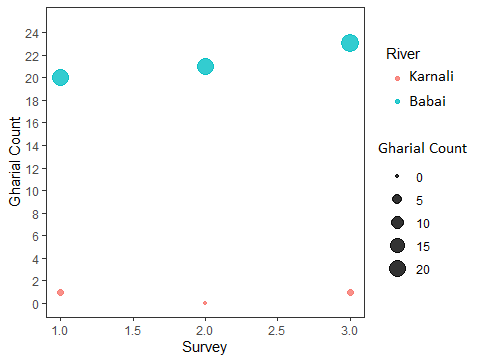


FIGURE S1 Figure showing the number of Gharials detected in different survey efforts.
